# Recent Views on Digestion

**Published:** 1870-06

**Authors:** J. R Bromwell


AMERICAN JOURNAL

OF

DENTAL SCIENCE.

Vol. IV. THIRD SERIES--JUNE, 1870. No. 2.

### ARTICLE I.

### *Recent Views on Digestion.*

BY J. R BROMWELL, D. D. S.

(*Concluded.*)

After the food has been trituated and disintegrated by the teeth, aided by the muscles of mastication, and thoroughly mixed with saliva, it becomes a soft pulpy mass, and is conducted by the involuntary muscular movements through the cardiac orifice into the stomach, there to be acted upon by the most important digestive fluid—viz. the gastric juice. Food after passing from the æsophagus enters the stomach in successive waves, and is immediatlely subjected to the peculiar peristaltic movement, the object of which is to thoroughly mix the gastric juice with the alimentary mass, and also to aid the solution of the latter by the gentle trituration it is subjected to.

The food after entering the stomach of man, is carried by the contractions and relaxations of the longitudinal and transverse fibres of the muscular coat, from the cardiac orifice down into the splenic extremity, and follows the great curvature towards the pyloric orifice. It then returns towards the small curvature, makes its appearance again at the aperture in its descent into the great curvature to perform similar revolutions.

These revolutions are performed in from one to three minutes ; the process is at first slow, and gradually increases, as chymefication advances. There is also towards the latter stage an increased impulse towards the pylorus. The food is not only subjected to the mechanical action of the stomach, but also to the liquifying, and catalytic action of the gastric juice. The gastric juice is secreted by the walls of the stomach, especially from the glands near the pyloric orifice. It is, in a pure state a clear, perfectly colorless acid fluid.

In place of the lactic acid, found by Lehmann, Bidder and Schmidt found hydrochloric. Lehmann also admits that hydrochloric acid is present in a small quantity, but with Robin and Verdeil, regards the acid reaction of the gastric juice, as due to lactic acid. Bernard states that hydrochloric acid, is only produced by the decomposition of some of the chlorides, which enter into the composition of the fresh gastric juice. The free acid of the juice is a very important ingredient, for if it be neutralized by an alkali, it fails to exert its solvent action upon food. The most important ingredient of the gastric juice is its organic compound pepsine.

It is regarded as one of the real, anatomical ingredients of the juice, and essential to its composition. It is the active digestive principle of the digestive fluid of the stomach. Gastric juice—like the saliva, is secreted in considerable quantity only under the stimulus of food. During the intervals of digestion, there is little or no gastric juice secreted, but immediately upon the introduction of food, the gastric juice is fully poured out, and in sufficient quantity to digest enough food to supply the waste of the system. The quantity of the secretion of the gastric juice varies according to the kinds of food, and the physical and mental condition of the man. Irritation of the temper, excitement, &c., will frequently diminish, or suspend the secretion of the gastric juice. Total quantity of gastric juice secreted daily in the human subject is 14 lbs. (av). This estimate, says Dr. Dalton, is not an exaggerated one.

Digestion is, strictly speaking, a chemical process, for food can be digested outside of the stomach by being exposed in a test tube to the action of pure gastic juice, which may be obtained from the stomach of a dog by means of an artificial gastric fistula, into the orifice of which a small silver canula is inserted so that the gastric fluid can escape. Food not only undergoes a liquifying process in the stomach, but all albuminoid substances are transformed by the catalytic action of the organic principle of the gastric fluid. For the active operation of this substance, it must be dissolved in an acid fluid, and be subjected to a certain degree of warmth. It is checked by any low or high degree of temperature. By the action of pepsine, all albuminoid substance without distinction are transformed into a new substance called albuminose. This substance is similar in its character to all organic compounds. It is not crystalizable, and contains nitrogen as one of its ultimate elements. This substance when in solution in gastric juice, inteferes with the operation of Tromer's test for grape sugar, and also with the mutual reaction of starch and iodine, for if starch be mixed with gastric juice containing albuminose, and iodine be added, it fails to produce the blue color. This does not take place when mixed with pure gastric juice. Another peculiar and remarkable property of the gastric juice, is its inaptitude for putrefaction. Dr. Dalton states that it may be kept for a long time in a common glass bottle without showing any signs of putrefaction. And again, although gastric readily attacks all food containing albumen, when introduced into the stomach it does not at all effect the walls of the stomach, for gastric juice is incapable of eroding living material. Dr. Dalton explains these singular and contradictory facts as follows: The process of digestion is not a simple solution, but a catalytic transformation of the alimentary substances, produced by contact with the pepsine of the gastric juice. We know that all the organic substances in the living tissues are constantly undergoing, in the process of nutrition, a series of catalytic changes, which are characteristic of the vital operations, and which are determined by the organized materials with which they are in contact, and by all other conditions present in the living organism. These changes, therefore, of nutrition, secretion, &c., necessarily exclude for the time all other catalyses and take precedence of them.

As soon as life departs, the walls of the stomach are at once attacked, and very often wholly destroyed.

The length of time for digestion varies according to the kinds of food, and is influenced, as before stated, by the mental as well as the physical condition of the man. For food to be digested properly it should never be taken, only when the appetite demands it; and it should be thoroughly masticated and mixed with the saliva. Many persons mistake appetite for hunger, and continue eating until the stomach is over-loaded, and the digestion of so large an amount of food being too much for the action of the stomach produces indigestion and finally, if the habit is continued, dyspepsia.

The food from the stomach, mixed with gastric juice, passes into the small intestine through the pyloric orifices. As before mentioned albuminoid substances are the only ones digested in the stomach. We therefore have left, as the remaining digestible ingredients of the food, the fats, starch and sugar. It has been stated by physiologists that cane sugar was digested in the stomach by the action of the gastric juice. This is now believed to be correct, for some of the most eminent physiologists of the present day, state that they have failed to detect the slightest trace of glucose, in the stomach of dogs fed upon sugar and then killed, or having gastric fistulæ. Cane sugar is therefore, either directly absorbed in the stomach, or passed into the intestinal canal to be acted upon by the fluids found therein. That starch is not digested in the stomach but in the intestines, is a matter in regard to which there is no longer any doubt.

The glands situated in the mucous membrane of the small intestine, follicles of Luberkuhn and Brunner's glands, secret the intestinal juice proper. This fluid has the property of converting starch into sugar. This effect may be produced outside of the intestine, by exposing starch in a test tube to the action of this fluid.

Dr. Dalton remarks that if a dog be fed with starch and meat, while some of the meat remains in the stomach from eight to ten hours, the starch begins to pass at once, after liquifaction, into the intestine, where it is converted into sugar and rapidly absorbed. The whole of the starch may be transformed into sugar, and absorbed in one hour.

In regard to the composition of the intestinal juice, there is nothing definite, owing to the fact that in the duodenum you find a mixture of three different fluids, viz: the bile the pancreatic juice and intestinal juice proper according to the experiments of Bidder and Schmidt. It resembles physically, the secretions of the mucous follicles of the mouth. It is a clear, colorless, glossy, viscid fluid with a distinct alkaline reaction, possessing the property of converting starch into sugar when mixed with it in a test tube, and kept at the temperature of 100° F. This fluid exists in small quantity in the intestine, although the follicles of Luberkuhn are very numerous. The next and last remaining ingredients of food that require digestion, are the fats or oily matters. These substances are only melted by the action of the gastric juice and the warmth of the stomach, and are set free from the vessels, fibres, &c., in which they are contained, and pass in this state into the duodenum to be acted upon by one of the intestinal juices, viz: the pancreatic fluid. Although mixing as the fats do with all three of the juices of the intestine, they are only acted upon by one of them. Bernard was the first to throw any light upon the digestion of fats, In the rabbit the biliary duct opens as usual just below the pylorus, while the pancreatic duct opens into the intestine some five or six inches below. Bernard fed there animals on fatty substance, then killed them and examined their small intestine; between the biliary and pancreatic ducts he found no chyle, but below the opening from the pancreas, the chyle was in abundance, proving clearly that fats are digested in the intestine by the action of the pancreatic fluid. Very soon after the oily matters enter into the small intestine and are mixed with pancreatic fluid, they loose their characteristic form, and are converted into a white, opaque emulsion known by the name of chyle. This emulsion undergoes no further change, but is at once absorbed. Pancreatic juice, as obtained by the experiments of Bidder and Schmidt, is a clear colorless slightly viscid fluid, with a very distinct alkaline reaction. It is composed of

| | | |
|---|---|---|
| Water, | | 900.76 |
| Organic Matter, | | 90.38 |
| Chloride of Sodium, | | 7.36 |
| Free Soda, | | 0.32 |
| Phosphate of Soda, | | 0.45 |
| Sulph. of Soda, | | 0.10 |
| Sulph. of Potassa, | | 0.02 |
| Combinations of | Lime, | 0.54 |
| | Magnesia, | 0.05 |
| | Oxide of Iron, | 0.02 |
| | Total | 1000.00 |

Like all the digestive fluids the most important ingredient of this juice is its organic matter or pancreatine. This substance is in greater abundance in this juice in proportion to its other ingredients, than is the organic matter of any other digestive fluid. It is similar to albumen in regard to its being coagulable by heat, but may be distinguished from it by its being precipitated by sulphate of magnesia. The action of this substance in all oleaginous matters is instantaneous and permanent.

The oils do not undergo chemical change by the action of this fluid, but are simply reduced to a state of perfect emulsion.

In this state the fat is capable of being absorbed, and therefore its digestion may be said to be accomplished. We have now followed all the different varieties of food through the process of digestion, and glanced hurridly at the nature and composition of the various digestive fluids, with the exception of the bile. Before speaking of this fluid, we will mention briefly some of the most important facts in regard to the anatomy of the organ by which it is secreted, the liver.

The liver is the largest glandular organ found in the body. It is situated immediately beneath the diaphragm, in the right hypochrondriac region and extends across the epigastrum into the left hyprochrondrium. Its weight, according to a statement made by Prof. Noel, is from 4 to 5 lbs.

Its transverse diameter is, according to Gray from 10 to 12 inches, antero-posterior diameter from 6 to 7 inches, and the back part of the right lobe (the thickest part) is 3 inches in thickness. Its upper surface is convex, covered by peritoneum, and is in relation with the diaphragm. This surface is divided into two unequal lobes by the suspensory or broad ligament.

The under surface is concave and in relation with the stomach, the duodenum, the hepatic flexor of the colon, the right kidney and supra renal capsul. This surface is also divided into two lobes, a right and left, by a longitudinal fissure.

The posterior border is rounded, broad, and connected with the diaphragm by the coronary ligament. The anterior border is sharp and thin, it is marked by a deep notch. The right extremity of the liver is thick and rounded, whilst the left is thin and flattened.

The liver has five ligaments, five fissures and five loes. The ligaments are the longitudinal, broad or suspensory, the two lateral, coronary and the round. The first four are formed of peritoneum, the fifth is a fibrous cord the result of the obliteration of the umbilical vein. The fissures are the longitudinal, the fissure of the ductus venosus, the transverse fissure, the fissure for the gall bladder, and the fissure for the vena cava.

The obes are, first and largest, the right; second. the left; third, lobulus quadratus; fourth the lobulus spigelii; fifth and finally, the lobulus caudatus.

The structure of the liver is held together by very fine areola tissue, it is composed of lobules agregated into lobes, of the ramifications of the portal vein, hepatic duct, hepatic artery, hepatic vein, lymphatics and nerves, the entire gland being enclosed by a fibrous, and serous coat. According to Gray, the lobules form the chief mass of the hepatic substance.

The function of the liver is mainly to secrete bile, and to produce sugar and fat. Bile is a dark golden brown, or greenish yellow fluid, is viscid and glutinous in consistency. Its reaction, says Dr. Dalton, is either neutral, or very slightly alkaline.

This fluid is depurative. If allowed to stay in the system for any length of time without undergoing transformation, it acts as a poison. It is to a certain extent antiseptic; when secreted in excess it acts as a purgative; if not in sufficient quantity costiveness and flatulence is the result. The coloring matter of bile is supposed to be its poisonous ingredient, owing to the fact that all the other ingredients of the bile are transformed or digested, reabsorbed and returned to the blood as hydrocarbon or fat; the biliverdin being discharged as fœcal matter. The function of the bile to a certain extent is digestion, it has the property of digesting the fatty acids, and promoting the absorption of fatty emulsions.

Some writers contend that bile is only an excrementitious substance, and has no important function to perform in the intestinal canal. This theory is disproved, for if we stop and consider for one moment the great size of this gland, we must conclude at once that it was intended to perform a more important function than that of simply excreting excrementitious products; and then again if the supply of bile be cut off from the intestinal canal, we have great emaciation, rapid degeneration of tissue and ultimately death.

The only ingredient of bile that may be considered as excrementitious is its coloring matter.

In conclusion, I will add that digestion is a complication of several processes, that take place successively in different parts of the alimentary canal.

The food on entering the mouth is subjected to the processes of mastication and insalivation, from there it passes into the stomach mixed with saliva to undergo the process of gastric digestion. That wholly or partially performed, it passes into the small intestine to undergo further digestion by the combined action of the gastric juice, which is still mixed with the food, and the various intestinal juices. The various indigestible and partially digested ingredients of food, are then passed through the ileo-cæcal valve into the large intestine to be finally discharged from the anus as fœces.

---

